# Activation of Trpv2 receptors increases the intravenous self-administration of low unit doses of cocaine in C3HeB/FeJ mice

**DOI:** 10.21203/rs.3.rs-10595093/v1

**Published:** 2026-09-01

**Authors:** Devon T. Applegate, Thomas R. Rahr, James David Jentsch

**Affiliations:** Binghamton University, State University of New York; Binghamton University, State University of New York; Binghamton University, State University of New York

**Keywords:** Reinforcement sensitivity, systems genetics, nucleus accumbens, individual differences, operant responding, O-1821

## Abstract

**Rationale:**

Genetic variation contributes to individual differences in cocaine self-administration. Systems-genetics analyses identified *Trpv2* as a candidate regulator of cocaine intake, with nucleus accumbens *Trpv2* mRNA expression being negatively correlated with cocaine intake. Whether Trpv2 functionally regulates cocaine reinforcement remains unknown.

**Objectives:**

We tested whether Trpv2 activation or inhibition affects cocaine self-administration.

**Methods:**

Male NOD/ShiLtJ and male and female C3HeB/FeJ mice were trained to self-administer cocaine under a FR1 schedule. During responding at 0.5 mg/kg/infusion, mice received vehicle, the Trpv2 agonist O-1821, tranilast, or the selective Trpv2 antagonist SET2. A separate male and female C3HeB/FeJ cohort underwent dose-response testing at 0.1, 0.5, and 1.0 mg/kg/infusion following O-1821 or vehicle, with sex evaluated as a between-subject factor.

**Results:**

NOD/ShiLtJ mice self-administered more cocaine than C3HeB/FeJ mice during acquisition. At a moderate dose of cocaine (0.5 mg/kg), neither Trpv2 activation nor antagonism altered cocaine intake in either strain. However, during dose-response testing, O-1821 increased cocaine intake at 0.1 mg/kg/infusion in C3HeB/FeJ mice in both sexes, along with significant sex-dependent reductions in cocaine intake with O-1821 at 0.5 and 1.0 mg/kg/infusion, respectively.

**Conclusions:**

Genetic background and baseline variation in *Trpv2* expression may contribute to individual differences in cocaine acquisition, whereas the acute effects of Trpv2 activation may depend on cocaine unit dose and sex. At low cocaine doses, Trpv2 activation may reduce reinforcement sensitivity and produce compensatory increases in intake. Trpv2 therefore warrants further investigation as a potential therapeutic target for substance use disorders.

## Introduction

Cocaine use disorder (CUD) remains a significant public health problem associated with substantial medical, psychiatric, and socioeconomic burden. Cocaine continues to contribute to overdose mortality in the United States, particularly in the context of polysubstance use involving drugs such as alcohol or synthetic opiates like fentanyl (Garnett and Minino 2024; Volkow and Blanco 2023). CUD is characterized by compulsive drug seeking and persistent drug use despite adverse consequences. Cocaine exerts its reinforcing effects, in part through inhibition of the plasmalemmal dopamine transporter, resulting in elevated extracellular dopamine within mesocorticolimbic circuitry, particularly the ventral tegmental area (VTA) and nucleus accumbens (NAc) (Giros et al. 1991; Hurd and Ungerstedt 1989; Koob and Volkow 2016; Volkow et al. 2009). Although dopaminergic signaling is implicated in cocaine reinforcement and reward, substantial individual variability exists in cocaine-related phenotypes, suggesting that additional modulatory systems contribute to cocaine responsivity and addiction vulnerability.

Genetic background strongly influences cocaine-related behaviors, including cocaine intravenous self-administration (IVSA), psychomotor sensitivity, and other reinforcement/reward-related phenotypes (Bagley et al. 2022; Dickson et al. 2025; Dickson et al. 2016; Roberts et al. 2018). Systems genetics approaches have therefore emerged as valuable tools for identifying molecular pathways associated with cocaine IVSA behavior. Recent work from our laboratory using the Hybrid Mouse Diversity Panel (HMDP) identified multiple loci associated with cocaine IVSA across genetically diverse inbred mouse strains (Khan et al. 2023). Genome-wide and transcriptome-wide association analyses converged to support transient receptor potential vanilloid 2 (Trpv2) as a candidate gene; Trpv2 expression in the nucleus accumbens is regulated in *cis* and is negatively correlated with cocaine intake and active lever responding across inbred strains. Colocalization analyses further supported Trpv2 as a candidate regulator of cocaine IVSA phenotypes.

Trpv2 encodes a calcium-permeable non-selective cation channel belonging to the transient receptor potential (TRP) vanilloid family (Caterina et al. 1999). Unlike classical ligand-gated neurotransmitter receptors, Trpv2 functions as a polymodal ion channel regulated by diverse physiological and biochemical stimuli, including lipid signaling molecules, intracellular signaling pathways, membrane stretch, and cannabinoid-related compounds (Lee 2019; Monet et al. 2009; Muraki et al. 2003; Perálvarez-Marín et al. 2013; Qin et al. 2008; Santoni et al. 2013; Shibasaki et al. 2010; Su et al. 2023; Zhang et al. 2022). Although Trpv2 has historically been studied primarily in peripheral tissues and immune-related processes (Iwata et al. 2009; Kanzaki et al. 1999; Link et al. 2010; Muraki et al. 2003; Yamashiro et al. 2010), emerging evidence suggests that Trpv2 is expressed within central reward circuitry and may influence dopaminergic neuronal activity. Recent studies have identified Trpv2 expression within dopaminergic neuronal populations of the substantia nigra and ventral tegmental area (Basu et al. 2025; Wang et al. 2024). Pharmacological activation of Trpv2 within the ventral midbrain dopaminergic nuclei has also been shown to alter locomotor behavior and striatal dopamine release (Basu et al. 2025).

Although our previous systems-genetics analyses identified an association between NAc *Trpv2* expression and cocaine self-administration (Khan et al. 2023), the mechanisms by which Trpv2 may influence cocaine reinforcement remain unclear. Since the NAc is the principal target of mesolimbic dopaminergic projections, variation in *Trpv2*/Trpv2 expression could influence dopamine-dependent reward processing. Trpv2 regulates intracellular calcium signaling and neuronal excitability, processes that influence neurotransmitter release, synaptic plasticity, and activity-dependent adaptations within reward-related circuitry (Higley and Sabatini 2012; Katz and Miledi 1967; Kauer and Malenka 2007; Malenka et al. 1988). Thus, variation in Trpv2 signaling may alter dopaminergic transmission and/or neuronal responsivity, ultimately contributing to individual differences in cocaine reinforcement.

The present study therefore examined whether pharmacological modulation of Trpv2 alters cocaine IVSA behavior in genetically distinct mouse strains that vary in their expression levels of *Trpv2* in the NAc (Khan et al. 2023); specifically, C3HeB/FeJ mice were selected due to their relatively higher expression, and NOD/ShiLtJ mice were selected for their relatively low expression (Khan et al. 2023). Cocaine IVSA procedures provide a widely used preclinical model for assessing drug reinforcement because drug delivery is contingent upon voluntary operant responding (Panlilio and Goldberg 2007; Wise and Koob 2014). Unlike non-contingent behavioral assays, IVSA paradigms allow for evaluation of cocaine intake, operant responding, and reinforcement sensitivity across experimental conditions (Roberts et al. 2018; Thomsen and Caine 2007). In our study, mice were administered pharmacological agents that either activate or inhibit Trpv2 prior to cocaine self-administration sessions, and cocaine intake and operant responding were assessed across experimental conditions. Based on our prior systems genetics findings demonstrating that elevated Trpv2 expression is associated with reduced cocaine intake, we reasoned that pharmacological activation of Trpv2 may phenocopy aspects of the high-Trpv2 expression phenotype (C3HeB/FeJ), whereas pharmacological inhibition may shift behavior toward that observed in low-Trpv2-expressing strains (NOD/ShiLtJ). Overall, we hypothesized that pharmacological activation of Trpv2 would reduce cocaine self-administration behaviors (cocaine infusions and active lever presses).

## Materials and Methods

### Subjects

Adult (8-week-old) male NOD/ShiLtJ (JAX Strain no. 001976) mice and adult male and female C3HeB/FeJ (JAX Strain no. 000658) mice obtained from The Jackson Laboratory (JAX; Bar Harbor ME). Female NOD/ShiLtJ mice were excluded due to age and body weight constraints, as well as strain-specific diabetic pathology, which precluded catheter implantation. Animals were shipped after implantation of indwelling jugular vein catheters performed at JAX and recovery. Catheters consisted of silicone tubing connected to a subcutaneous vascular access button (Instech Laboratories, Plymouth Meeting PA), which allowed repeated intravenous drug delivery, while minimizing handling stress during behavioral testing in IVSA sessions.

Upon arrival, mice were allowed to acclimate before any behavioral testing began. Catheter patency and general health were monitored and maintained throughout the experiment. Catheters were flushed daily with 0.05 mL sterile 0.9% saline (Vetivex^®^, Dechra Veterinary Products, Overland Park, KS) and subsequently filled with a heparin-glycerol lock solution (0.01 mL volume, 500 units/mL concentration; SAI Infusion Technologies, Lake Villa, IL). Patency was verified prior to initiation of pharmacological treatment experiments by infusions of propofol (~ 0.02 mL volume, 10 mg/mL concentration; Zoetis, NJ) and again upon completion to ensure eligibility for inclusion in data analyses. A total of 64 mice were acquired (Experiments 1a–1b: n = 40 [NOD/ShiLtJ: 24 males; C3HeB/FeJ: 8 males, 8 females]; Experiment 2: n = 24 [C3HeB/FeJ: 12 males, 12 females]). Mice were housed under standard laboratory conditions with *ad libitum* access to food and water, except during daily testing sessions. All procedures were conducted in accordance with The Guide for the Care and Use of Laboratory Animals, 8th Edition (National Research Council (U.S.). Committee for the Update of the Guide for the Care and Use of Laboratory Animals. et al. 2011) and were approved by Binghamton University’s Institutional Animal Care and Use Committee.

#### Drugs and Vehicles

Cocaine hydrochloride (Sigma-Aldrich; St. Louis, MO) was dissolved in sterile 0.9% saline. The infusion concentration (0.84 mg/mL) produced a freebase dose of 0.5 mg/kg/infusion delivered in a volume of 0.67 mL/kg/infusion, consistent with prior intravenous self-administration studies from our laboratory (Bagley et al. 2022). All acquisition and pharmacological testing in experiments 1a-1b used a unit dose of 0.5 mg/kg/infusion. This unit dose was selected because it was the cocaine dose used in the prior HMDP systems-genetics study that identified Trpv2 as a candidate regulator of cocaine self-administration (Bagley et al. 2022; Khan et al. 2023). For dose-response testing in experiment 2, cocaine unit doses of 0.1, 0.5, and 1.0 mg/kg/infusion were tested to span a range of reinforcing magnitudes.

O-1821 (cannabidiorcol; Cayman Chemical; Ann Arbor, MI) was used as a selective pharmacological agonist of Trpv2 based on prior evidence demonstrating preferential activation of Trpv2 relative to other TRP channels (Laragione et al. 2015). O-1821 is structurally related to cannabinoids and was originally characterized in early phytocannabinoid pharmacology work (Vree et al. 1972). As relatively few *in vivo* behavioral studies of O-1821 are available (Laragione et al. 2015; Laragione et al. 2023), doses were selected conservatively and tested in logarithmic progression (3.2 and 10 mg/kg) based on our own pilot tolerability assessments. O-1821 was supplied as a 10 mg/ml solution in acetonitrile. The acetonitrile vehicle was removed via centrifugal evaporation, and the compound was then reconstituted in minute quantities of 100% dimethyl sulfoxide (DMSO; Thermo Fisher Scientific, Waltham, MA) followed by dilution with sterile saline to the final working concentrations used for administration (0.32 mg/mL and 1 mg/mL).

Tranilast (Cayman Chemical; Ann Arbor, MI) was used as a pharmacological inhibitor of Trpv2 signaling. Although initially described as a direct Trpv2 antagonist (Iwata et al. 2009; Shiota et al. 2010; Yatsunami et al. 2000), more recent work suggests that tranilast modulates Trpv2 activity indirectly via reduction of reactive oxygen species and redox-dependent channel activation (Fricke et al. 2025). tranilast is clinically approved in Japan and the Republic of Korea for the treatment of asthma and hypertrophic scarring and has been widely investigated in inflammatory and fibrotic disease models (Chuang et al. 2023). Doses of 10 and 30 mg/kg were selected based on prior *in vivo* studies demonstrating central pharmacological activity while minimizing nonspecific behavioral effects (Shiota et al. 2010; Yatsunami et al. 2000).

SET2 (Cayman Chemical; Ann Arbor, MI) was used as a more selective Trpv2 inhibitor to distinguish Trpv2-dependent effects from broader off-target, redox- or anti-inflammatory mechanisms associated with tranilast exposure (Chai et al. 2019; Zhong et al. 2022). Doses of 10 and 30 mg/kg were chosen to parallel tranilast while maintaining behavioral tolerability. SET2 was administered during experiment 1b pharmacological testing following re-establishment of baseline cocaine intake.

O-1821, tranilast or SET2 were initially dissolved in 100% DMSO and were subsequently diluted stepwise with sterile saline to achieve a final vehicle concentration of 5% DMSO prior to administration. SET2 consistently yielded a precipitate during the elution process, so it was administered as a suspension. The 5% DMSO vehicle was selected to maintain drug solubility while minimizing injection volume and nonspecific behavioral effects. Vehicle control conditions were included in all experiments and were reintroduced when necessary to re-establish baseline responding between pharmacological testing blocks. All drugs were administered systemically prior to IVSA sessions at time points selected to coincide with peak behavioral effects during testing.

#### Intraperitoneal Injections

All pharmacological agents (except IVSA cocaine) and vehicle solutions were administered via intraperitoneal (i.p.) injection prior to behavioral testing. Injections were performed 30 min before placement into the operant chambers. Injection volumes were delivered at a 10 mL/kg equivolume basis across all treatment conditions. Injection volumes were weight-adjusted for each subject and did not exceed 0.35 mL per mouse. All drug solutions were prepared fresh each experimental day to ensure stability and dosing consistency. Animals were briefly handled for injections and returned to their home cages during the pretreatment interval before transfer to the testing room. Vehicle control injections (5% DMSO in sterile saline) were administered using the same procedures, timing, and injection volumes as drug treatments.

At the time these experiments were conducted, detailed pharmacokinetic data were not available for O-1821, tranilast, or SET2 in mice. Our 30-min pretreatment interval was selected based on pharmacokinetic studies of structurally related lipophilic cannabinoid compounds. Following intraperitoneal administration, cannabinoids such as Δ9-tetrahydrocannabinol and cannabidiol reach peak brain concentrations within approximately 30–120 min (Deiana et al. 2012; Grotenhermen 2003; Hložek et al. 2017; Huestis 2007). The 30-min pretreatment interval was therefore chosen to ensure drug administration occurred within the expected window of peak central drug exposure during behavioral testing. Notably, pharmacokinetic work published after completion of these experiments demonstrated that the selective Trpv2 antagonist SET2 reaches peak plasma concentrations within minutes of intraperitoneal administration and remains detectable for at least 2 h post-injection (Bartosova et al. 2025), supporting the pretreatment interval selected for the present behavioral experiments.

#### Cocaine IVSA Testing

IVSA sessions were conducted in standard mouse operant conditioning chambers (Med Associates; St. Albans, VT) housed within sound-attenuating cubicles and equipped with ventilation fans that provided constant background white noise. Each chamber was fitted with two retractable ultrasensitive levers positioned on the left and right ends of the front wall of the chamber, a house light, five horizontally arranged stimulus lights located on the rear wall, and a syringe pump system and tether system for intravenous drug delivery. The apparatus, infusion hardware, and behavioral procedures were identical to those previously described, ensuring methodological consistency across studies from our laboratory (Bagley et al. 2022; Khan et al. 2023), except that the maximum number of infusions permitted per session was increased during dose-response testing (65 ◊ 100). Syringe pumps delivered infusions through tubing connected to a fluid swivel mounted above the chamber, allowing unrestricted movement of the animals during testing. Assignment of the active lever (left vs. right) was counterbalanced across strain and sex. Testing chambers were assigned in a non-randomized manner to minimize testing multiple animals from the same strain in the same chamber.

Testing occurred during the light phase of a 12:12 h light/dark cycle at the same time each day. Sessions began with activation of the house light and illumination of the five rear stimulus lights. No priming infusions were delivered at the start of sessions. Responding was maintained under a fixed-ratio 1 (FR1) schedule of reinforcement. Each response on the active lever resulted in delivery of one cocaine infusion paired with discrete visual cues. Infusions were followed by a 20-s timeout period during which the house light flashed, and the stimulus lights were extinguished. Responses on the active lever during the timeout period were recorded but had no programmed consequences. Responses on the inactive lever were recorded throughout the session but were never reinforced. For experiment 1a and 1b, sessions terminated after 65 infusions were earned or 2 h elapsed, whichever occurred first. For experiment 2 (dose-response testing), the infusion cap was increased to 100 infusions to prevent ceiling effects and to allow full characterization of responding at the lowest cocaine unit dose (0.1 mg/kg/infusion).

#### Acquisition of cocaine self-administration

Mice underwent 10 consecutive daily acquisition sessions at 0.5 mg/kg/infusion of cocaine. Acquisition criteria were based on multiple behavioral indicators of stable and goal-directed responding, including escalation of cocaine intake across sessions, stable infusion and response patterns across consecutive sessions and reliable discrimination (≥ 70%) between active and inactive levers. Animals meeting these criteria advanced to the pharmacological testing portion of the experiments. This acquisition period ensured the establishment of robust operant responding prior to experimental manipulations. These acquisition criteria are consistent with established intravenous self-administration procedures used to define stable drug-reinforced responding (Panlilio and Goldberg 2007; Slosky et al. 2022; Thomsen and Caine 2007).

### Pharmacological testing under established self-administration

Following acquisition, mice underwent pharmacological testing using within-subject Latin-square designs to control for order effects and individual differences in baseline cocaine intake. Drug test sessions were separated by 48-h washout intervals to minimize potential carryover effects (Panlilio and Goldberg 2007; Slosky et al. 2022; Thomsen and Caine 2007). Between drug test sessions, animals completed an additional self-administration session without pretreatment to re-establish stable baseline responding. Consequently, the testing sequence followed a treatment day ◊ washout day ◊ baseline re-establishment day ◊ next treatment day schedule, resulting in approximately 72 h between successive drug treatment sessions. This approach ensured that each pharmacological condition was evaluated against a stable and recently verified baseline level of cocaine intake, while allowing each animal to serve as its own control.

Experiment 1a examined selective Trpv2 activation and non-selective antagonism during ongoing cocaine IVSA. Animals were tested under the following pretreatment conditions: Vehicle (5% DMSO), O-1821 (3.2 mg/kg), O-1821 (10 mg/kg), tranilast (10 mg/kg), or tranilast (30 mg/kg). As previously mentioned, these doses were selected based on prior literature and pilot work demonstrating behavioral efficacy without nonspecific locomotor impairment. This experiment evaluated how pharmacological modulation of Trpv2 influences ongoing cocaine reinforcement under stable FR1 conditions.

Experiment 1b aimed to test selective Trpv2 antagonism during ongoing cocaine IVSA. To further evaluate the effects of an alternative, more selective Trpv2 antagonist, animals were tested following a 30-min pretreatment of Vehicle (5% DMSO), SET2 (10 mg/kg), or SET2 (30 mg/kg). Vehicle testing was intentionally reintroduced at the start of this experiment to re-establish baseline intake following the prior pharmacological testing block.

Experiment 2 aimed to determine whether Trpv2 activation alters cocaine reinforcement sensitivity. A new set of mice were first trained to self-administer the 0.5 mg/kg/infusion unit dose, before completing IVSA sessions across multiple cocaine unit doses: 0.1 mg/kg/infusion, 0.5 mg/kg/infusion, or 1.0 mg/kg/infusion. Sessions were conducted following 30-min pretreatment with vehicle or O-1821 (3.2 or 10 mg/kg) using a within-subject design. This experiment allowed assessment of potential left- or right-shifts in the cocaine dose-response curve, providing insight into changes in cocaine reinforcing efficacy and potency.

#### Primary Behavioral Measures

Primary dependent variables included: 1) Cocaine infusions earned, 2) Active lever responses, and 3) lever discrimination (active / inactive total responses). These measures permitted dissociation of drug intake, motivation for cocaine reinforcement, nonspecific behavioral activation, and operant discrimination.

#### Statistical analyses

Acquisition curves were analyzed using repeated-measures ANOVA with session/day as a within-subject factor and strain as a between-subject factor where appropriate. Behavioral data during the pharmacological testing portion of experiments were analyzed using repeated-measures ANOVAs with Greenhouse-Geisser correction when sphericity assumptions were violated. Planned paired comparisons were further conducted to test *a priori* hypotheses regarding Trpv2 modulation of cocaine reinforcement. Where appropriate, strain (C3HeB/FeJ vs. NOD/ShiLtJ) was included as a between-subjects factor. Analyses were conducted using GraphPad Prism (Version 10.24.1) and SPSS (Version 29). Statistical significance was set at α = 0.05. When significant main effects or interactions were detected, Tukey’s multiple-comparisons test was used for post hoc pairwise comparisons. Planned a priori comparisons were additionally evaluated using two-tailed tests. Sex was not analyzed as an independent factor in the combined-strain analyses for Experiments 1a-1b because both sexes were not represented in both strains. For Experiment 2, cocaine infusions were analyzed using a mixed three-way repeated measures ANOVA with cocaine unit dose (0.1, 0.5, 1.0 mg/kg/infusion) and O-1821 pretreatment (vehicle, 3.2 mg/kg, 10 mg/kg) as within-subject factors and sex as a between-subject factor. Secondary exploratory analyses were further conducted within each cocaine unit dose to better characterize sex-dependent O-1821 effects across the dose-response curve.

## Results

### Acquisition of cocaine self-administration and lever discrimination

During the acquisition period for experiments 1a-1b, all 16 C3HeB/FeJ mice and 18 of 24 NOD/ShiLtJ mice met acquisition criteria for intravenous cocaine self-administration under a fixed-ratio 1 schedule of reinforcement (Fig. 1, Supplementary table 1). More specifically, in the mice included in experiment 1a and1b, a total of 6 of 24 NOD/ShiLtJ mice failed to meet acquisition criteria and were excluded from further testing; as a result, final sample sizes for experiments 1a-1b were n = 16 C3HeB/FeJ (8 male, 8 female) mice and n = 18 NOD/ShiLtJ male mice. Dose-response testing in experiment 2 was conducted in a separate cohort of C3HeB/FeJ mice (n = 24; n = 12 per sex); of these, three animals failed to meet acquisition criteria and were excluded (2 males, 1 female), yielding a final sample size of n = 21.

Acquisition performance was examined across all animals in the study, as testing was identical prior to the start of the challenge studies. Across the 10 acquisition sessions, cocaine intake progressively increased as animals learned the operant response contingency (Fig. 1). A two-way repeated-measures ANOVA including session and strain revealed a significant main effect of session (F(9,342) = 7.41, p < 0.0001). A significant main effect of strain was also observed (F(1,38) = 4.48, p = 0.0409), together with a significant Session × Strain interaction (F(9,342) = 3.90, p < 0.0001), consistent with our prior reports (Bagley et al. 2022; Khan et al. 2023), NOD/ShiLtJ mice exhibited higher levels of cocaine self-administration relative to C3HeB/FeJ mice across acquisition (Fig. 1, Supplementary table 1). To confirm acquisition in the cohort used for cocaine dose-response testing (experiment 2), acquisition curves were analyzed separately in this subset of C3HeB/FeJ mice. Repeated-measures ANOVA revealed a significant effect of session/day (F(2.98, 68.60) = 11.75, p < 0.0001), demonstrating progressive increases in cocaine intake across acquisition sessions in this cohort as well (see Supplementary table 1).

### Effects of Trpv2 activation with O-1821 under moderate-cocaine dose testing conditions

To determine whether Trpv2 activation alters cocaine reinforcement under moderate cocaine dose testing conditions, mice were pretreated with vehicle or the Trpv2 agonist O-1821 (3.2 or 10 mg/kg) prior to IVSA sessions conducted at a cocaine unit dose of 0.5 mg/kg/infusion (Fig. 2). At the 65-infusion session cap, some C3HeB/FeJ mice reached the cap across vehicle and O-1821 conditions (vehicle: 0/16; O-1821 3.2 mg/kg: 0/16; O-1821 10 mg/kg: 1/16), whereas this was more common in NOD/ShiLtJ mice (vehicle: 4/18; O-1821 3.2 mg/kg: 3/18; O-1821 10 mg/kg: 4/18). To directly compare strain sensitivity, a two-way repeated-measures ANOVA including both strains was conducted. This analysis revealed no significant main effect of treatment (F(2,64) = 2.024, p = 0.1405), no main effect of strain (F(1,32) = 0.199, p = 0.6584), and no Treatment × Strain interaction (F(2,64) = 0.224, p = 0.7999). In C3HeB/FeJ mice only, cocaine intake did not differ significantly across treatment conditions. A repeated-measures one-way ANOVA revealed no significant effect of O-1821 dose on the number of cocaine infusions earned (F(1.861, 27.92) = 1.987, p = 0.1584). Planned within-subject comparisons confirmed that neither the 3.2 mg/kg dose (t(15) = 1.39, p = 0.1845) nor the 10 mg/kg dose (t(15) = 1.75, p = 0.1003) differed significantly from vehicle pretreatment. A similar pattern was observed in NOD/ShiLtJ mice; repeated-measures ANOVA indicated no effect of O-1821 dose on cocaine intake (F(1.906, 32.40) = 0.537, p = 0.581), and planned paired comparisons revealed no significant differences between vehicle and either O-1821 dose. Collectively, these findings indicate that acute Trpv2 activation does not alter cocaine self-administration when cocaine is available at a moderate reinforcing dose under standard moderate cocaine unit dose testing conditions. Analysis of active lever responding produced a similar pattern. A two-way ANOVA revealed no significant Treatment × Strain interaction (F(2,64) = 1.057, p = 0.3534), no main effect of treatment (F(2,64) = 2.151, p = 0.1247), and no main effect of strain (F(1,32) = 0.078, p = 0.7818), indicating that O-1821 did not significantly alter operant responding across strains (Supplementary table 2).

### Effects of Trpv2 inhibition with tranilast on cocaine self-administration

To evaluate whether Trpv2 inhibition alters cocaine reinforcement, mice were tested following pretreatment with tranilast (10 or 30 mg/kg; Fig. 3). At the 65-infusion session cap, a small number of C3HeB/FeJ mice reached the cap across vehicle and tranilast conditions (vehicle: 0/16; tranilast 10 mg/kg: 1/16; tranilast 30 mg/kg: 0/16), while a larger subset of NOD/ShiLtJ mice reached the cap in each condition (vehicle: 4/18; tranilast 10 mg/kg: 3/18; tranilast 30 mg/kg: 3/18). A combined strain analysis revealed no significant Treatment × Strain interaction (F(2,64) = 1.559, p = 0.218) and no main effect of strain (F(1,32) = 0.637, p = 0.431). A trend toward a main effect of treatment was observed (F(2,64) = 3.00, p = 0.0568). In C3HeB/FeJ mice, repeated-measures ANOVA did not reveal a significant overall effect of tranilast on cocaine intake (F(1.59, 23.87) = 1.68, p = 0.210). However, planned within-subject comparisons revealed a modest but statistically significant increase in cocaine intake following the 10 mg/kg dose relative to vehicle (t(15) = 2.396, p = 0.030), whereas the 30 mg/kg dose did not differ from vehicle (t(15) = 0.502, p = 0.623). In contrast, tranilast did not significantly alter cocaine intake in NOD/ShiLtJ mice. The repeated-measures ANOVA indicated no significant treatment effect (F(1.30, 22.08) = 1.78, p = 0.197), and paired comparisons did not reveal significant differences at either dose (10 mg/kg: t(17) = 1.309, p = 0.208; 30 mg/kg: t(17) = 1.427, p = 0.172). Together, these findings indicate that Trpv2 inhibition with tranilast does not produce a robust omnibus effect across strains on cocaine self-administration, although a dose-specific increase in intake was observed in C3HeB/FeJ mice at the lower tranilast dose.

### Effects of selective Trpv2 antagonism with SET2

To determine whether selective antagonism of Trpv2 alters cocaine reinforcement, mice were next tested following pretreatment with SET2 (10 or 30 mg/kg; Fig. 4). During SET2 testing, the 65-infusion cap was reached by 2/16 C3HeB/FeJ mice under vehicle and SET2 10 mg/kg conditions and by 4/16 C3HeB/FeJ mice under the SET2 30 mg/kg condition. In NOD/ShiLtJ mice, 4/18 animals reached the cap in each condition. Combined strain analysis revealed a trend towards a significant Treatment × Strain interaction (F(2,64) = 2.654, p = 0.0781) and no main effects of treatment or strain. In C3HeB/FeJ mice, repeated-measures ANOVA revealed a trend toward a main effect of treatment (F(1.864, 27.95) = 3.375, p = 0.0517), though planned comparisons did not reveal significant differences between vehicle or either dose (10 mg/kg: p = 0.2491; 30 mg/kg: p = 0.1357). In NOD/ShiLtJ mice, SET2 produced no detectable effects on cocaine intake (F(1.489, 25.31) = 0.2947, p = 0.6825), and paired comparisons versus vehicle were not significant. Thus, selective Trpv2 antagonism with SET2 did not significantly alter cocaine self-administration under moderate dose conditions, although strain-dependent trends may be observed.

Similarly, analyses of active lever responding failed to reveal significant treatment effects within either strain (Supplementary table 4), and lever preference ratios remained stable across treatment conditions (Supplementary tables 3–5), indicating preserved discrimination between active and inactive levers throughout testing.

#### Effects of Trpv2 activation on the cocaine dose-response function

Two features of our initial experimental design may have limited our ability to detect Trpv2 ligand effects. First, a number of animals reached the 65-infusion cap, producing a potential ceiling effect. Second, we only tested one dose of cocaine (0.5 mg/kg), which is around the peak of the dose-response curve. To address this, we conducted an additional experiment with a higher infusion cap (100) and across a range of cocaine doses (0.1–1.0 mg/kg/infusion). Mice were first trained to reliably respond at 0.5 mg/kg/infusion across 10 days of acquisition prior to dose-substitution testing, after which 0.1, 0.5, and 1.0 mg/kg/infusion doses of cocaine were made available and pretreatment with the Trpv2 agonist O-1821 (Fig. 5) was conducted. Despite increasing the cap, a number of mice still reached the session cap at the lowest cocaine unit dose: at 0.1 mg/kg/infusion, 8/21 mice reached the 100-infusion cap following vehicle pretreatment, whereas 15/21 mice reached the cap following either 3.2 or 10 mg/kg O-1821. In contrast, no mice reached the 100-infusion cap at 0.5 or 1.0 mg/kg/infusion under any pretreatment condition. A two-way repeated-measures ANOVA revealed a significant main effect of cocaine dose (F(1.236, 24.72) = 275.8, p < 0.0001). A significant main effect of O-1821 dose was also observed (F(1.932, 38.63) = 3.433, p = 0.0439), but the O-1821 × cocaine dose interaction was not significant (F(2.571, 51.41) = 2.061, p = 0.1255).

Analysis of active lever responding revealed a robust main effect of cocaine dose (F(1.132, 22.65) = 275.1, p < 0.0001), with a trend toward a main effect of O-1821 dose (F(1.978, 39.55) = 3.155, p = 0.0541); there was no significant interaction between these two variables (F(2.199, 43.98) = 2.266, p = 0.1110). Lever preference ratios remained high across all conditions, and inactive lever responding was low and unaffected by treatment (p > 0.1), indicating preserved operant discrimination between active and inactive levers. Although the overall O-1821 × cocaine dose interaction was not significant, planned within-dose analyses were conducted a priori because our hypothesis specifically predicted that Trpv2 modulation would be most evident at lower reinforcing cocaine doses along the dose-response function, rather than uniformly across all doses.

Planned analyses revealed that, at the lowest cocaine unit dose (0.1 mg/kg/infusion), there was a significant effect of O-1821 treatment (F(1.904, 38.07) = 3.335, p = 0.0485) on cocaine infusions earned. Paired comparisons demonstrated that pretreatment with 3.2 mg/kg O-1821 significantly increased cocaine infusions relative to vehicle (t(20) = 2.508, p = 0.0209), whereas the 10 mg/kg dose produced a trend toward increased intake (t(20) = 2.064, p = 0.0522). In contrast, O-1821 did not significantly alter cocaine intake at either the moderate (0.5 mg/kg/infusion; F(1.892, 37.84) = 0.3550, p = 0.6918) or high cocaine dose (1.0 mg/kg/infusion; F(1.721, 34.42) = 0.5207, p = 0.5718). Additional analyses of active lever responding indicated a trend toward a treatment effect following O-1821 administration in C3HeB/FeJ mice (F(1.8, 27.04) = 3.31, p = 0.056) (Supplementary table 4). However, these response changes were not accompanied by significant changes in cocaine infusions earned outside the low-dose condition, indicating that Trpv2 activation did not broadly alter cocaine intake across the dose-response function.

Importantly, inactive lever responding remained low, and lever preference ratios remained high across all cocaine doses and treatment conditions (Supplementary tables 3–5), indicating that the increase in cocaine intake observed following O-1821 administration at 0.1 mg/kg/infusion reflected altered cocaine-directed responding rather than impaired operant discrimination or nonspecific behavioral activation. Collectively, the pooled analyses indicate that Trpv2 activation increased cocaine self-administration under conditions of low reinforcement strength, consistent with a possible rightward shift in the cocaine dose-response function. Although no overall treatment effects were detected at 0.5 or 1.0 mg/kg/infusion, subsequent exploratory analyses examined whether opposing sex-dependent responses contributed to these pooled results.

#### Sex-dependent effects of O-1821 across the dose-response function

To further examine whether the effects of O-1821 differed by sex across the cocaine dose-response function, cocaine infusions were analyzed using a mixed three-way repeated-measures ANOVA with cocaine unit dose and O-1821 pretreatment as within-subject factors and sex as a between-subject factor (Fig. 6). The Cocaine Dose × O-1821 × Sex interaction approached significance, *F*(2.245, 42.646) = 2.899, *p* = 0.060. The Cocaine Dose × O-1821 interaction was not significant, *F*(2.245, 42.646) = 2.213, *p* = 0.116, nor were the O-1821 × Sex interaction, *F*(1.917, 36.424) = 1.038, *p* = 0.362, or the Cocaine Dose × Sex interaction, *F*(1.192, 22.646) = 0.645, *p* = 0.439. The main effect of sex approached significance, *F*(1, 19) = 3.509, *p* = 0.077, whereas significant main effects of cocaine dose, *F*(1.192, 22.646) = 270.848, *p* < 0.001, and O-1821, *F*(1.917, 36.424) = 3.342, *p* = 0.048, were observed.

Secondary within-dose analyses were conducted to further characterize this pattern (Fig. 6). At 0.1 mg/kg/infusion, there was again a significant main effect of O-1821, *F*(2, 38) = 3.313, *p* = 0.0472, consistent with the pooled analysis presented in Fig. 5 and reflecting increased cocaine intake following O-1821 pretreatment in both sexes. At 0.5 mg/kg/infusion, a significant O-1821 × Sex interaction was observed, *F*(1.989, 37.80) = 4.231, *p* = 0.0221, together with a significant main effect of sex, *F*(1, 19) = 6.953, *p* = 0.0163. This interaction was characterized by reduced cocaine intake following O-1821 pretreatment in females, whereas intake in males was unaffected. At 1.0 mg/kg/infusion, the O-1821 × Sex interaction was again significant, *F*(1.895, 36.01) = 6.194, *p* = 0.0055, whereas the main effect of sex approached significance, *F*(1, 19) = 4.292, *p* = 0.0521. At this dose, O-1821 was associated with reduced cocaine intake in males but trended towards an increase in intake in females. Collectively, these exploratory analyses indicate that the effects of O-1821 across the cocaine dose-response function differed by sex, particularly at the moderate and high cocaine unit doses. These opposing sex-dependent responses appear to have obscured treatment-related effects at 0.5 and 1.0 mg/kg/infusion in the pooled analyses shown in Fig. 5.

## Discussion

### Reinforcement Sensitivity and Dose-Response Modulation

The present study investigated whether pharmacological modulation of Trpv2 influences cocaine reinforcement-related behavior in mouse strains previously identified as differing in endogenous Trpv2 expression and cocaine self-administration phenotypes. Strain-related differences were evident during acquisition, with NOD/ShiLtJ mice exhibiting greater cocaine self-administration than C3HeB/FeJ mice, consistent with prior systems-genetics findings associating lower nucleus accumbens Trpv2 expression with greater cocaine intake (Khan et al. 2023). Contrary to our original hypothesis, pharmacological activation or antagonism of Trpv2 did not produce robust or consistent changes in cocaine self-administration between strains at the moderate cocaine unit dose (0.5 mg/kg/infusion). However, during dose-response testing conducted in C3HeB/FeJ mice (strain that previously was characterized with high *Trpv2* expression in the NAc), Trpv2 activation via O-1821 increased cocaine intake at the lowest unit dose examined, whereas further analyses revealed sex-dependent O-1821 effects at the moderate and high cocaine unit doses. Together, these findings suggest that the behavioral consequences of Trpv2 signaling may depend on genetic background, cocaine dose and biological sex.

Dose-response testing revealed that Trpv2 activation significantly increased cocaine self-administration at the lowest cocaine unit dose. This could be consistent with a rightward shift in the cocaine dose-response function, such that greater intake was required to compensate for reduced sensitivity to cocaine’s reinforcing effects. Importantly, the pooled analyses did not identify overall O-1821 effects at the moderate or high cocaine unit doses. However, the exploratory sex analyses indicated that this apparent absence of treatment effects reflected opposing responses in males and females rather than a uniform lack of O-1821 sensitivity. Thus, Trpv2 activation may also alter the relationship between cocaine dose and behavioral output in a sex-dependent manner.

One possible explanation for these sex-dependent effects is that males and females occupy different baseline positions along the cocaine dose-response function. If female C3HeB/FeJ mice are more sensitive to cocaine’s reinforcing effects, their dose-response function may be relatively left-shifted compared with males. Under this model, an O-1821-induced reduction in cocaine sensitivity could become evident at a lower cocaine unit dose in females, consistent with the reduction in intake observed at 0.5 mg/kg/infusion. In males, a comparable reduction may emerge only at the higher 1.0 mg/kg/infusion dose. Thus, the reductions observed at different unit doses may reflect a related change in cocaine sensitivity expressed from different baseline positions along the dose-response function. The modest increase in female intake at 1.0 mg/kg/infusion indicates that the findings are not fully explained by a simple parallel rightward shift, however, and suggests that additional sex-dependent processes may shape the behavioral consequences of Trpv2 activation at higher cocaine doses.

Gonadal hormone signaling represents one potential contributor to these sex-dependent effects. Estradiol has been shown to enhance the acquisition and maintenance of cocaine self-administration in female rodents, although its influence depends on dose, sex, hormonal context, and the specific phase of cocaine-taking behavior being assessed (Hu and Becker 2008; Jackson et al. 2006; Lynch et al. 2001; Zhao and Becker 2010). Estradiol may also interact directly or indirectly with Trpv2 signaling. Structural and functional studies indicate that estradiol can potentiate ligand-induced Trpv2 activation, while *Trpv2* mRNA expression in several mouse brain regions varies across the estrous cycle and is elevated during proestrus relative to other cycle stages (Kumar et al. 2018; Su et al. 2023).These observations raise the possibility that hormonal state influences Trpv2 expression or channel responsiveness and thereby contributes to sex-dependent behavioral effects of O-1821. However, estrous-cycle stage and circulating hormone levels were not measured in the present study, and the available evidence does not establish estradiol-dependent regulation of Trpv2 specifically within the nucleus accumbens circuitry controlling cocaine self-administration. Accordingly, this mechanism remains speculative and will require direct testing.

The interpretation of these findings is further informed by the training history and reinforcement schedule under which behavior was acquired. All animals were initially trained to self-administer cocaine at 0.5 mg/kg/infusion under a fixed-ratio 1 (FR1) schedule, a condition that establishes a stable reference level of reinforcement efficacy (Panlilio and Goldberg 2007). As a result, subsequent behavior during dose-response testing reflects deviations from this learned reinforcement baseline. When cocaine was available at a lower unit dose (0.1 mg/kg/infusion), each infusion delivered less effective than the training condition, leading animals to increase responding to compensate for reduced magnitude of effect. In contrast, at the higher unit dose (1.0 mg/kg/infusion), each infusion produced greater effects than the training condition, resulting in reduced responding due to increased per-infusion efficacy. This bidirectional adjustment is consistent with well-established evidence that animals regulate drug intake to maintain a preferred level of drug effect under fixed-ratio schedules (Norman and Tsibulsky 2006; Panlilio and Goldberg 2007).

Critically, because behavior was assessed under an FR1 schedule, the present findings primarily reflect changes in reinforcement sensitivity under low-effort conditions. Under FR1 schedules, animals can readily compensate for changes in reinforcement efficacy by adjusting response rate. In contrast, more demanding schedules, such as progressive ratio procedures, impose increasing response requirements and are more sensitive to changes in motivational processes and reinforcing efficacy (Oleson et al. 2011; Richardson and Roberts 1996). Thus, while the present data demonstrate that Trpv2 signaling alters cocaine reinforcement sensitivity and intake, future studies incorporating higher-effort schedules will be important for determining whether Trpv2 also modulates the motivational properties of cocaine.

### Reinforcement vs. Performance and Experimental Design Considerations

An important consideration in interpreting these findings is the distinction between reinforcement-related processes and performance-related effects. IVSA behavior reflects both the motivational properties of the drug and the animal’s ability to execute the operant response (Panlilio and Goldberg 2007; Thomsen and Caine 2007). Several lines of evidence indicate that the effects of Trpv2 modulation observed here reflect changes in reinforcement sensitivity rather than nonspecific alterations in performance. Lever discrimination remained high across all subsequent experimental testing, including pharmacological manipulations and dose-response procedures (see below), demonstrating preserved task performance and reinforcement specificity throughout the experiment. Lever preference ratios were not significantly altered by O-1821, tranilast, or SET2 treatment (all treatment effects p > 0.3; Supplementary table 3). However, a consistent main effect of strain was observed across pharmacological testing experiments, indicating that NOD/ShiLtJ mice exhibited lower preference for the active lever than C3HeB/FeJ mice despite comparable overall levels of cocaine intake (Supplementary table 3). This pattern suggests modestly weaker operant discrimination in NOD/ShiLtJ mice while preserving cocaine-directed responding.

The within-subject design used during pharmacological testing allowed each animal to serve as its own control, thereby reducing the influence of stable between-subject differences in baseline cocaine intake (Panlilio and Goldberg 2007; Thomsen and Caine 2007). Accordingly, the effects observed following O-1821 administration reflect acute changes relative to each animal’s established baseline, whereas the strain differences observed during acquisition likely represent broader, trait-like differences in cocaine self-administration, strain differences that are consistent with our prior systems-genetics findings linking higher Trpv2 expression to reduced cocaine intake (Khan et al. 2023). Together, these findings suggest that Trpv2 is associated with strain-related baseline differences in cocaine reinforcement (between-subject effects) and contributes to dynamic modulation of reinforcement sensitivity under pharmacological manipulation (within-subject effects), which vary according to cocaine dose and biological sex.

### Trpv2, Individual Differences, and Stage-Dependent Contributions to Cocaine-Related Behavior

Our previous systems genetic studies indicated that relatively high *Trpv2* expression is associated with reduced cocaine self-administration during acquisition (Khan et al. 2023). Here, we found that pharmacological activation of Trpv2 increased cocaine intake under conditions of reduced reinforcement magnitude, particularly in a strain documented to express high *Trvp2* mRNA in the nucleus accumbens, while its effects at moderate and high cocaine unit doses varied by sex. The strain differences observed during acquisition likely reflect stable, trait-like differences in cocaine reinforcement sensitivity, instrumental learning and/or motivational tone associated with endogenous variation in Trpv2 expression. In contrast, the present pharmacological experiments examined the acute consequences of manipulating Trpv2 signaling after cocaine-taking behavior had already been established. Since the neural processes governing acquisition are not identical to those maintaining well-learned drug-taking behavior, Trpv2 may contribute differently across stages of cocaine exposure. Early cocaine use is thought to depend heavily on reinforcement learning and reward valuation, whereas extended self-administration increasingly reflects maintenance of a learned reinforcement set point and the emergence of more habitual behavioral processes and, perhaps, the development of a level of tolerance (Belin et al. 2008; Everitt and Robbins 2016). Accordingly, Trpv2 may contribute differently to baseline differences in cocaine reinforcement sensitivity and to dynamic modulation of reinforcement processes across the progression of active cocaine IVSA.

### Interpreting the Absence of Selective Trpv2 Antagonist Effects

An unexpected finding of the present study was the relative absence of behavioral effects following selective Trpv2 antagonism with SET2 under the 0.5 mg/kg/infusion cocaine condition. SET2 did not significantly alter cocaine intake, active lever responding, or lever discrimination at this moderate cocaine unit dose. However, SET2 was not evaluated across the cocaine dose-response function. As the effects of Trpv2 activation emerged specifically at the lowest cocaine unit dose examined, it remains possible that Trpv2 antagonism would similarly produce detectable effects under low-dose conditions. Therefore, the present findings should be interpreted as evidence that SET2 did not alter established cocaine self-administration under the specific testing conditions used, rather than as evidence that endogenous Trpv2 signaling does not influence cocaine reinforcement more generally.

One possibility is that endogenous Trpv2 signaling exerts relatively little influence over cocaine self-administration at the moderate unit dose tested here but may become more behaviorally relevant when the reinforcing effects of cocaine are weaker. In this framework, basal Trpv2 signaling may not exert sufficient influence over ongoing reinforcement processes for pharmacological blockade to produce measurable behavioral consequences. In contrast, exogenous activation of Trpv2 may recruit signaling mechanisms that are not normally engaged to a substantial degree during stable cocaine self-administration, thereby revealing a latent capacity of this system to modulate reinforcement-related behavior. Such a model would be consistent with the observation that O-1821 altered responding under low-dose cocaine conditions, whereas SET2 produced little detectable behavioral effect under moderate dose conditions. Under this interpretation, Trpv2 may function as a modulatory system whose influence on cocaine reinforcement becomes most apparent when channel activity is experimentally enhanced rather than inhibited.

Alternatively, methodological considerations may have contributed to the absence of robust antagonist effects. At the time these experiments were conducted, detailed pharmacokinetic and tissue-distribution data for SET2 were not available. Subsequent characterization has suggested that SET2 exhibits relatively limited tissue penetration following systemic administration despite detectable plasma exposure (Bartosova et al. 2025). Consequently, it remains possible that the doses, pretreatment intervals, or route of administration employed in the present study did not achieve sufficient central target engagement to fully antagonize Trpv2 signaling within neural circuits regulating cocaine reinforcement. This possibility is particularly relevant given that Trpv2 is expressed across multiple peripheral and central tissues, making systemic exposure an imperfect indicator of effective receptor occupancy within reward-related brain regions.

Importantly, the absence of SET2 effects does not necessarily argue against a role for Trpv2 in cocaine-related behavior. Rather, the combined agonist and antagonist findings suggest that Trpv2-dependent modulation may be more readily observed when channel activity is experimentally enhanced than when basal signaling is inhibited. Future studies incorporating direct measures of target engagement, alternative routes of administration, region-specific manipulations, or genetic approaches will be important for determining whether endogenous Trpv2 signaling contributes to cocaine reinforcement under conditions not captured by the present experimental design.

### Limitations and Future Directions

Several limitations should be considered when interpreting the present findings. First, behavioral testing was conducted exclusively under fixed-ratio 1 (FR1) schedules, which primarily assess reinforcement processes under relatively low response requirements. Consequently, the present experiments cannot determine whether Trpv2 also influences motivation to obtain cocaine under higher-effort conditions. Second, the current experiments relied upon systemic pharmacological manipulation and therefore do not identify the specific neural circuits through which Trpv2 influences cocaine-related behavior. An additional limitation concerns the analysis of sex. Because NOD/ShiLtJ mice were represented only by males, sex could not be evaluated independently in the combined-strain analyses. The sex-dependent effects identified in Experiment 2 were derived from a single C3HeB/FeJ cohort and from secondary within-dose analyses; therefore, they should be considered exploratory until replicated in an independent, fully sex-balanced cohort. Estrous-cycle stage and circulating gonadal hormone concentrations were also not assessed, preventing determination of whether hormonal state affected the cocaine or Trvp2 ligand response in females. Finally, while the present findings demonstrate effects on cocaine self-administration, they do not address whether Trpv2 similarly contributes to other cocaine-related phenotypes, including cocaine reward, cue-induced drug seeking, or relapse-like behavior. Future studies employing progressive-ratio procedures, region-specific manipulations, and complementary behavioral approaches will be important for further defining the role of Trpv2 in addiction-related processes.

## Conclusions

Taken together, the present findings identify Trpv2 as a novel regulator of cocaine reinforcement sensitivity and provide the first functional evidence that pharmacological manipulation of Trpv2 signaling alters cocaine self-administration behavior. Strain differences during acquisition were consistent with the possibility that genetic background and baseline variation in *Trpv2* expression contribute to individual differences in cocaine-taking behavior. Contrary to our original hypothesis of a uniform reduction in cocaine intake, the effects of Trpv2 activation depended on cocaine unit dose and sex. O-1821 increased intake at the lowest cocaine unit dose across sexes, possibly due to compensatory responding following reduced cocaine sensitivity, whereas sex-dependent reductions emerged at higher unit doses, with decreased intake in females at 0.5 mg/kg/infusion and in males at 1.0 mg/kg/infusion. These findings extend prior systems genetics observations linking *Trpv2* expression to cocaine intake and support a model in which Trpv2 contributes to individual differences in cocaine reinforcement sensitivity. More broadly, the present results establish a foundation for future studies investigating how Trpv2-dependent signaling influences reinforcement learning, neuroplasticity, and vulnerability to cocaine use disorder across different stages of addiction, and support further investigation of Trpv2 as a potential therapeutic target for substance use disorders, while emphasizing the need to determine how its behavioral effects vary across drug dose, biological sex, genetic background, and stage of addiction.

## Supplementary Material

Supplementary Files

This is a list of supplementary files associated with this preprint. Click to download.

- SupplementalTablesIVSA.docx

## Figures and Tables

**Figure 1:**
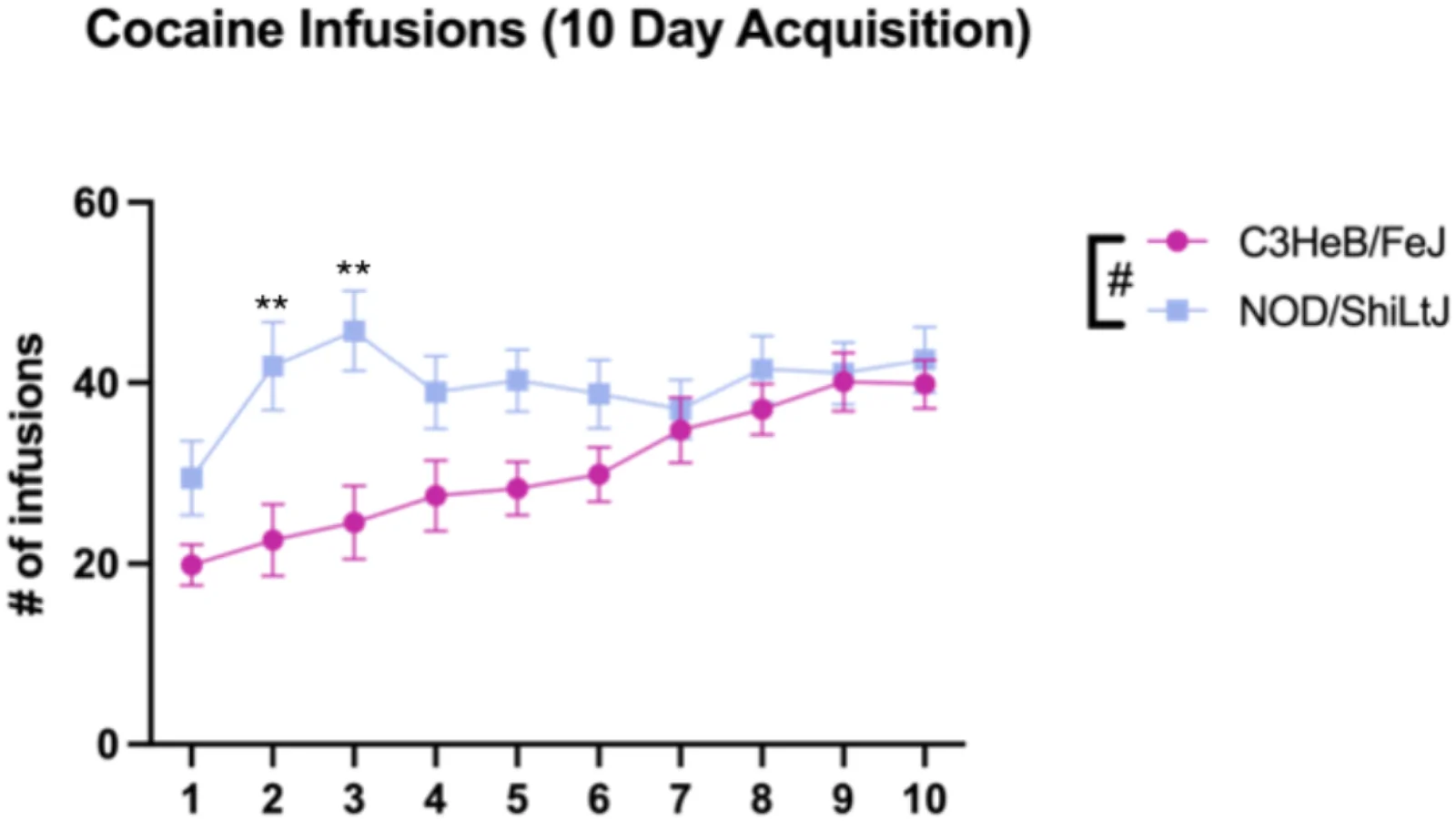
Acquisition of cocaine self-administration across 10 training sessions in C3HeB/FeJ and NOD/ShiLtJ mice Cocaine infusions earned across 10 acquisition sessions under a fixed-ratio 1 schedule at 0.5 mg/kg/infusion. Infusions increased across sessions, and NOD/ShiLtJ mice exhibited greater cocaine intake than C3HeB/FeJ mice during acquisition. Data are presented as mean ± SEM. C3HeB/FeJ, *n* = 16; NOD/ShiLtJ, *n* = 24. #*p* < 0.05, main effect of strain; ** *p* < 0.01, between-strain difference at the indicated sessions.

**Figure 2:**
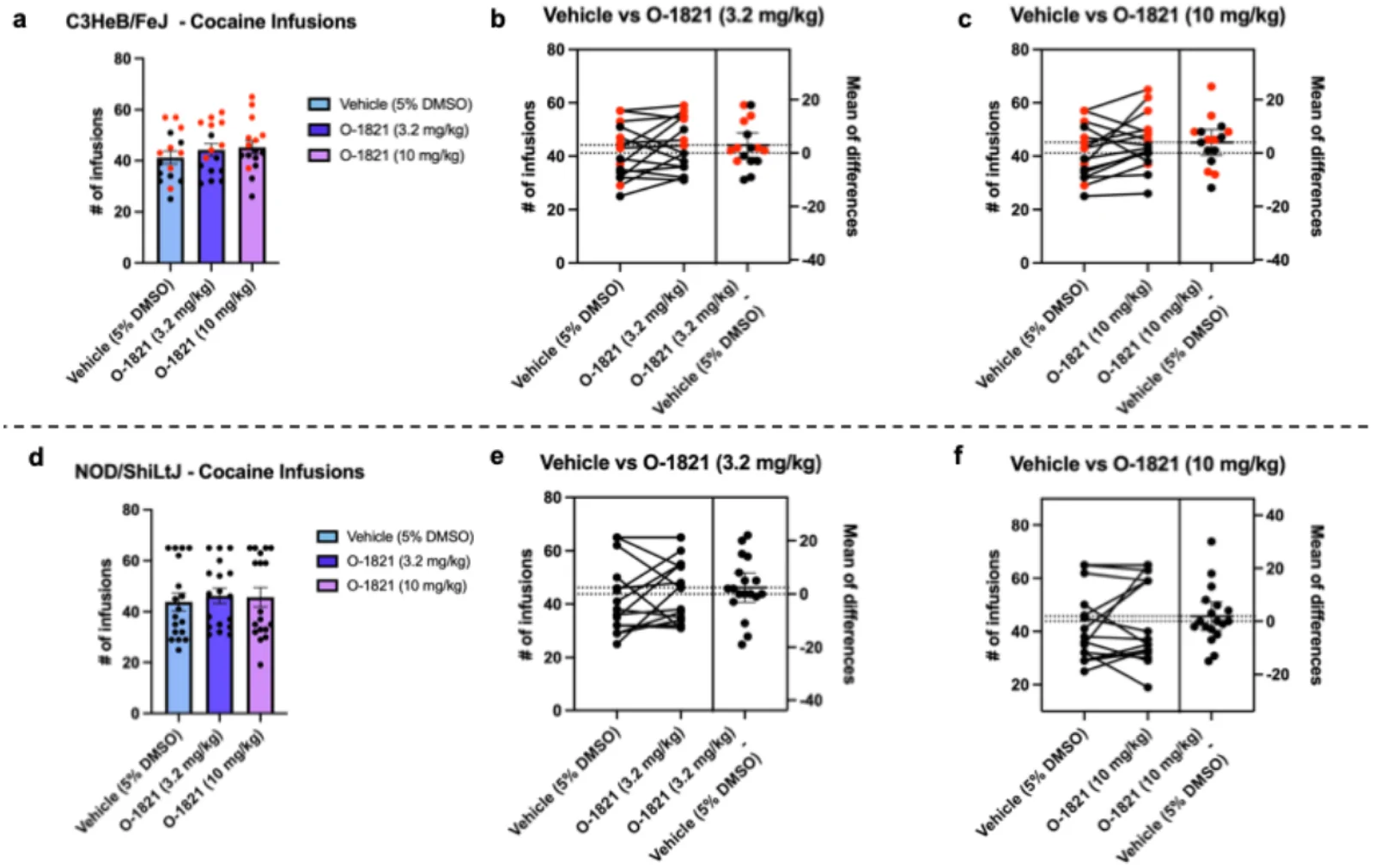
Effects of Trpv2 activation with O-1821 on cocaine self-administration at 0.5 mg/kg/infusion in C3HeB/FeJ and NOD/ShiLtJ mice Cocaine infusions earned following pretreatment with vehicle, 3.2 mg/kg O-1821, or 10 mg/kg O-1821. (a) Cocaine infusions in C3HeB/FeJ mice across treatment conditions. (b, c) Within-subject comparisons between vehicle and 3.2 mg/kg O-1821 (c) or 10 mg/kg O-1821 (c), with corresponding difference plots. (c) Cocaine infusions in NOD/ShiLtJ mice across treatment conditions. (e, f) Within-subject comparisons between vehicle and 3.2 mg/kg O-1821 (e) or 10 mg/kg O-1821 (f), with corresponding difference plots. Data are presented as individual values with mean ± SEM. C3HeB/FeJ, n = 16; NOD/ShiLtJ, n = 18. Red symbols denote females, and black symbols denote males.

**Figure 3:**
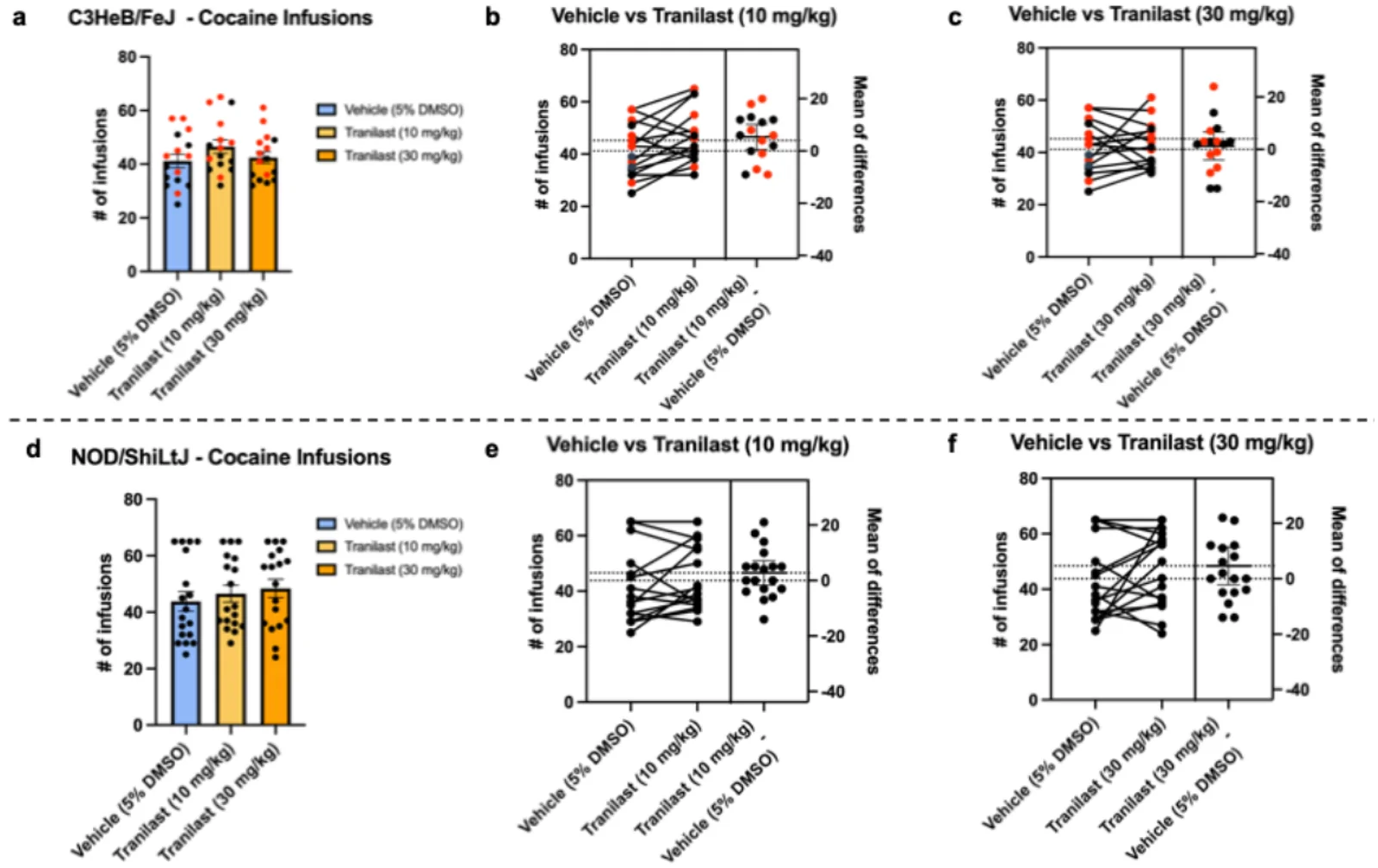
Effects of Trpv2 inhibition with tranilast on cocaine self-administration at 0.5 mg/kg/infusion in C3HeB/FeJ and NOD/ShiLtJ mice Cocaine infusions earned following pretreatment with vehicle, 10 mg/kg tranilast, or 30 mg/kg tranilast. (a) Cocaine infusions in C3HeB/FeJ mice across treatment conditions. (b, c) Within-subject comparisons between vehicle and 10 mg/kg tranilast (b) or 30 mg/kg tranilast (c), with corresponding difference plots. (d) Cocaine infusions in NOD/ShiLtJ mice across treatment conditions. (e, f) Within-subject comparisons between vehicle and 10 mg/kg tranilast (e) or 30 mg/kg tranilast (f), with corresponding difference plots. Data are presented as individual values with mean ± SEM. C3HeB/FeJ, *n* = 16; NOD/ShiLtJ, *n* = 18. Red symbols denote females, and black symbols denote males.

**Figure 4:**
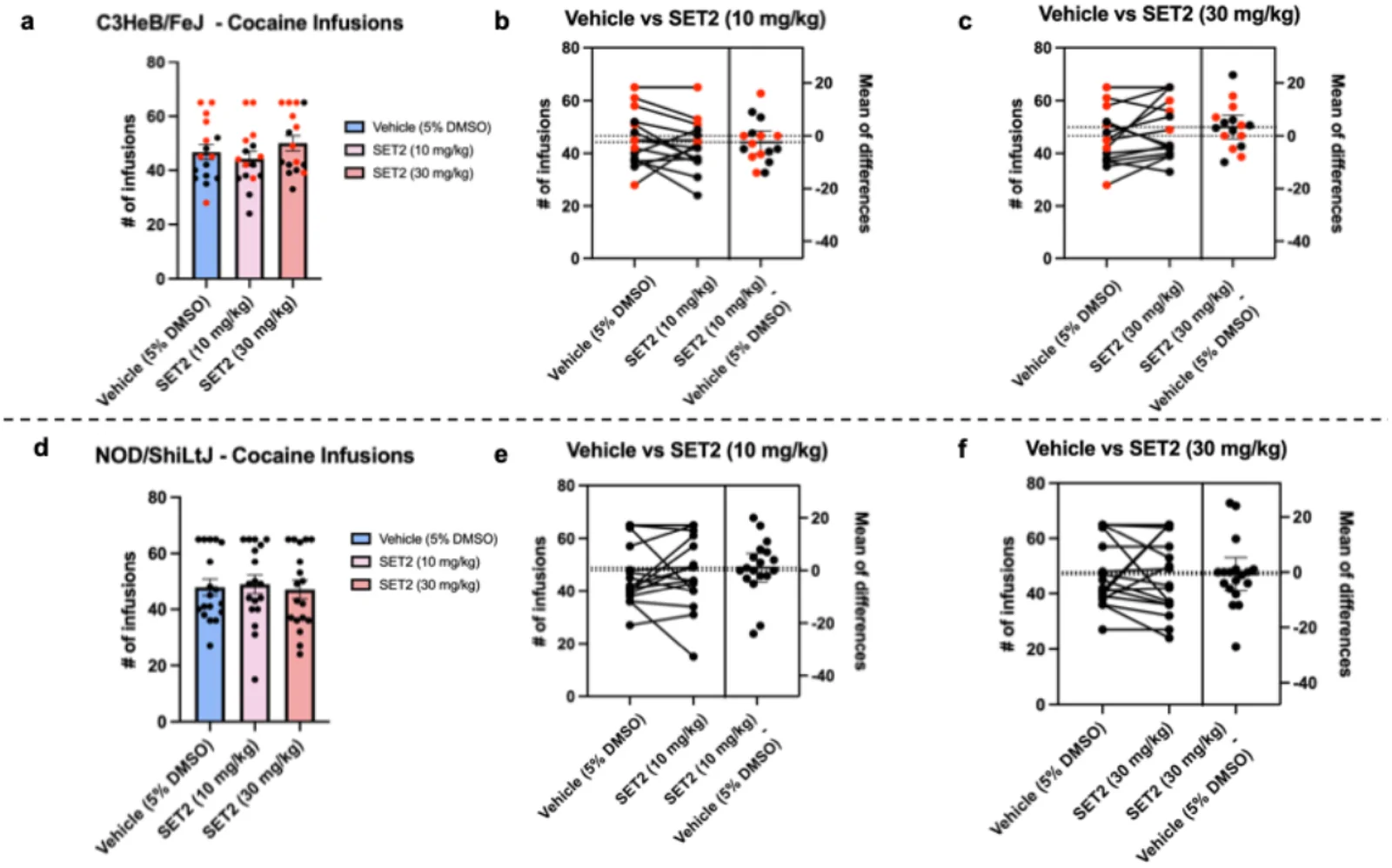
Effects of selective Trpv2 inhibition with SET2 on cocaine self-administration at 0.5 mg/kg/infusion in C3HeB/FeJ and NOD/ShiLtJ mice Cocaine infusions earned following pretreatment with vehicle, 10 mg/kg SET2, or 30 mg/kg SET2. (a) Cocaine infusions in C3HeB/FeJ mice across treatment conditions. (b, c) Within-subject comparisons between vehicle and 10 mg/kg SET2 (b) or 30 mg/kg SET2 (c), with corresponding difference plots. (d) Cocaine infusions in NOD/ShiLtJ mice across treatment conditions. (e, f) Within-subject comparisons between vehicle and 10 mg/kg SET2 (e) or 30 mg/kg SET2 (f), with corresponding difference plots. Data are presented as individual values with mean ± SEM. C3HeB/FeJ, *n* = 16; NOD/ShiLtJ, *n* = 18. Red symbols denote females, and black symbols denote males.

**Figure 5:**
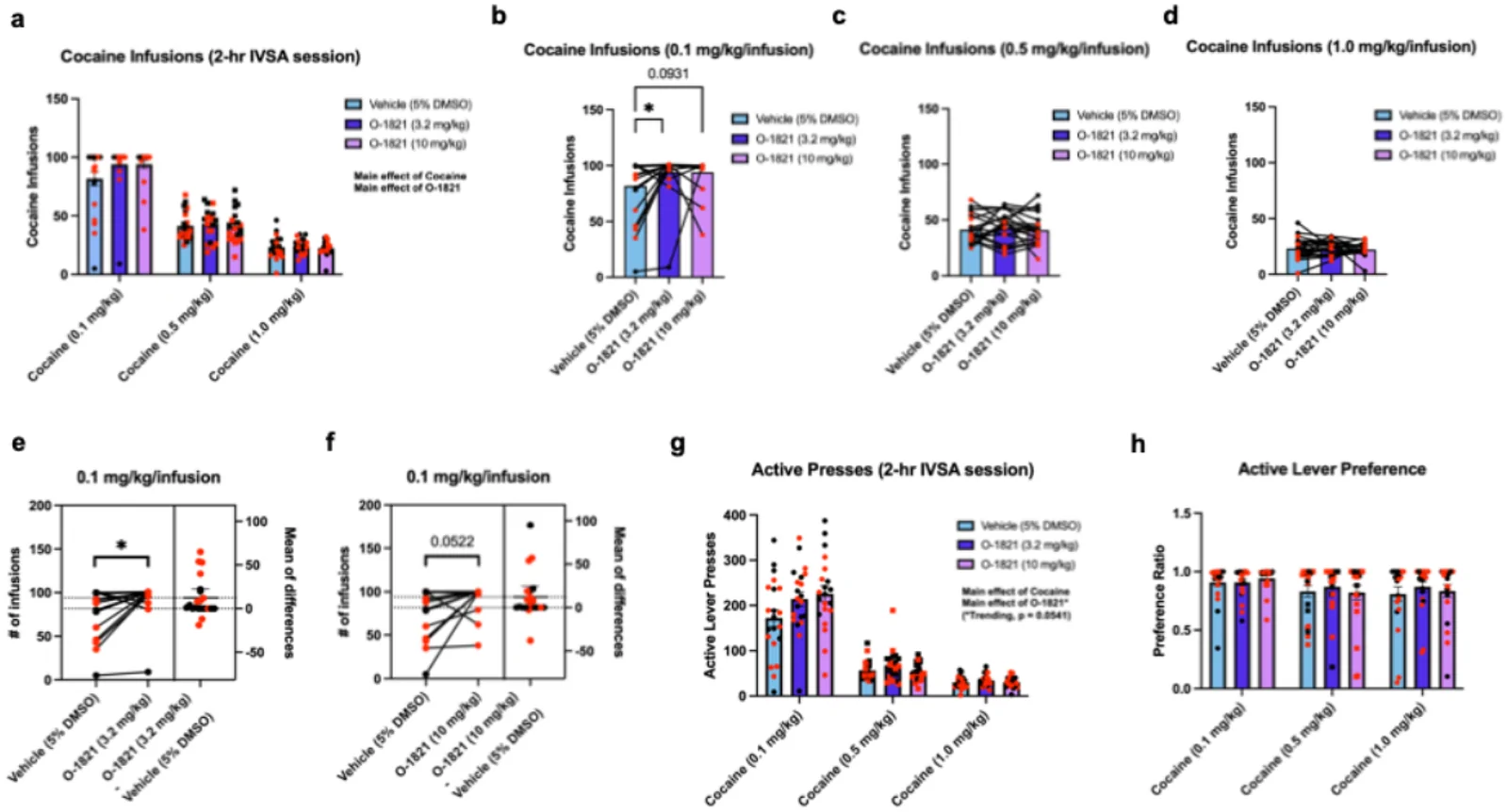
Effects of Trpv2 activation with O-1821 across the cocaine dose-response function in C3HeB/FeJ mice Cocaine self-administration was assessed during 2-h intravenous self-administration sessions at cocaine unit doses of 0.1, 0.5, and 1.0 mg/kg/infusion following pretreatment with vehicle, 3.2 mg/kg O-1821, or 10 mg/kg O-1821. (a) Cocaine infusions earned across cocaine doses and pretreatment conditions. (b–d) Cocaine infusions earned at 0.1 mg/kg/infusion (b), 0.5 mg/kg/infusion (c), and 1.0 mg/kg/infusion (d). (e, f) Within-subject comparisons between vehicle and 3.2 mg/kg O-1821 (e) or 10 mg/kg O-1821 (f) at 0.1 mg/kg/infusion, with corresponding difference plots. (g) Active-lever responses across cocaine doses and pretreatment conditions. (h) Active-lever preference ratios across cocaine doses and pretreatment conditions. Data are presented as individual values with mean ± SEM. *n* = 21 mice (10 males and 11 females). Red symbols denote females, and black symbols denote males. * *p* < 0.05 versus vehicle; the exact *p* value is shown for the comparison that did not reach statistical significance.

**Figure 6:**
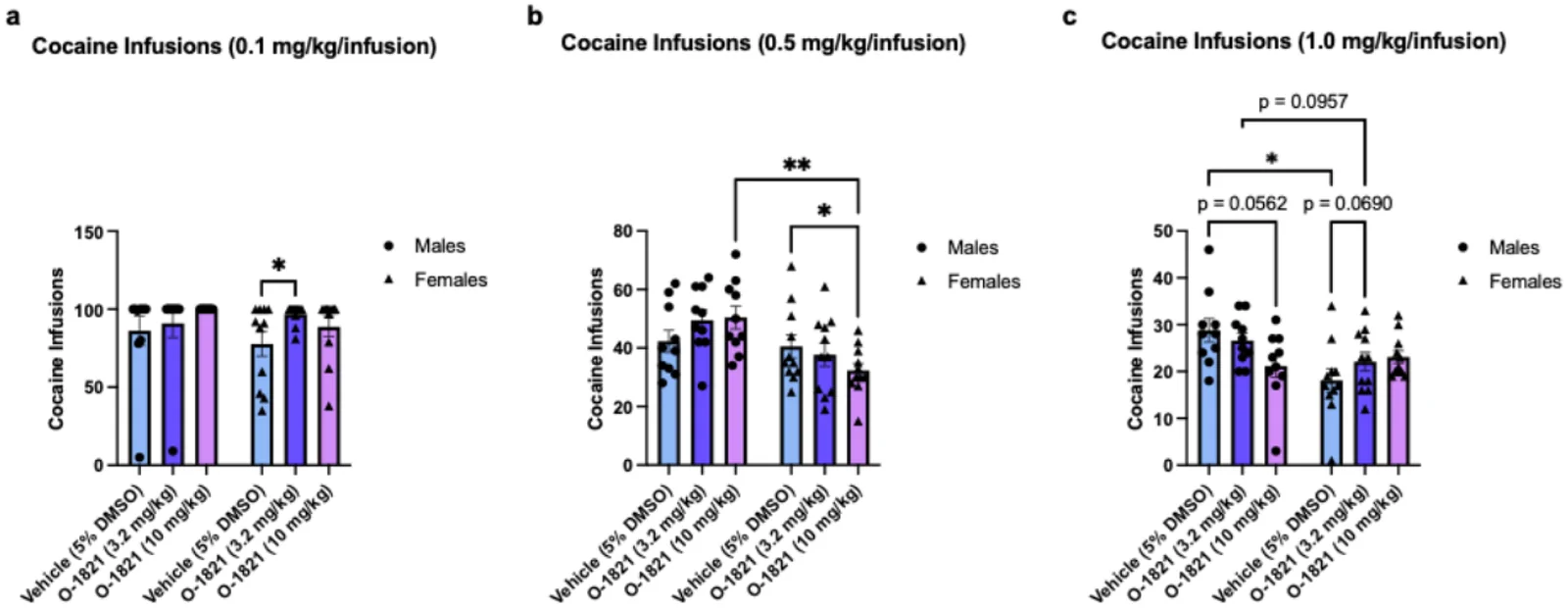
Sex-dependent effects of O-1821 across the cocaine dose-response function in C3HeB/FeJ mice Cocaine infusions earned following pretreatment with vehicle, 3.2 mg/kg O-1821, or 10 mg/kg O-1821 during intravenous self-administration sessions conducted at cocaine unit doses of (a) 0.1, (b) 0.5, and (c) 1.0 mg/kg/infusion. At 0.1 mg/kg/infusion, O-1821 increased cocaine intake across sexes. At 0.5 and 1.0 mg/kg/infusion, significant O-1821 × Sex interactions reflected differing treatment-response patterns in males and females. Data are presented as individual values with mean ± SEM. Circles denote males and triangles denote females; n = 21 mice total (10 males and 11 females). * *p* < 0.05; ** p < 0.01. Exact *p* values are shown for comparisons that approached, but did not reach, statistical significance.

## Data Availability

The datasets generated and analyzed during the current study will be deposited in The Open Repository @ Binghamton (The ORB) and made publicly available upon publication.
